# Prognostic factors associated with locally advanced gastric cancer patients treated with neoadjuvant chemotherapy followed by surgical resection

**DOI:** 10.18632/oncotarget.20660

**Published:** 2017-09-06

**Authors:** Yongkun Sun, Lin Yang, Chengfeng Wang, Dongbing Zhao, Jianqiang Cai, Wenbin Li, Wen Zhang, Jing Huang, Aiping Zhou

**Affiliations:** ^1^ National Cancer Center/Cancer Hospital, Chinese Academy of Medical Sciences & Peking Union Medical College, Beijing, China

**Keywords:** prognostic factors, locally advanced gastric cancer, neoadjuvant chemotherapy, gastrectomy

## Abstract

In this retrospective study, we analyzed prognostic factors associated with survival outcomes in 73 locally advanced gastric cancer patients treated with neoadjuvant chemotherapy (NAC) followed by surgical resection. Median disease-free survival (DFS) for 64 patients that received R0 resection was 685 days, whereas median overall survival (OS) for 73 patients was 930 days. Multivariate analysis demonstrated that post-treatment nodal stages (P = 0.002), nervous invasion (P = 0.0492) and serum CA199 levels (P = 0.0398) were independent prognostic factors for DFS. Nodal stages (P = 0.0007), presence of nervous invasion (P = 0.0259) and non-radical resection (P = 0.0165) were independent prognostic factors for OS. These results indicate that post-treatment nodal stages, neural invasion and serum CA199 levels are all associated with poor DFS. Moreover, post-treatment nodal stage, resection type and neural invasion status are independent prognostic factors for OS.

## INTRODUCTION

Gastric cancer ranks fifth among most malignant cancers and third among cancer related deaths worldwide [1]. China has the highest incidence of gastric cancer accounting for 35% of total gastric cancer cases worldwide with a high mortality rate of 25.16 cases per 100,000 [2]. Prognosis of locally advanced gastric cancer is poor with a 5-year overall survival (OS) rate of20-30% for surgery-only patients [3]. Neoadjuvant chemotherapy (NAC) is preferred for locally advanced gastric cancer patients since the release of the MRC Adjuvant Gastric Infusional Chemotherapy (MAGIC) trial results [4]. Many studies demonstrate that NAC reduces the size of gastric cancer lesions thereby decreasing tumor staging and increasing the chances for radical resection and survival while decreasing post-operative complications [5–9]. However, standard regimen and courses of NAC are not yet established and prognostic factors associated with survival outcomes for patients treated with NAC followed by surgery remain unclear.

Therefore, we analyzed the relevant prognostic factors associated with survival by reviewing medical records of 73 locally advanced gastric cancer patients that were treated with neoadjuvant chemotherapy followed by curative-intent surgery. Our analysis included the prognostic status of pathological response to NAC and post-therapy node status (Nodal stage or lymph node metastasis ratio).

## RESULTS

### Patient and treatment characteristics

Table 1 shows patient and treatment characteristics. The median patient age was 53.0 years (range:32–77 yrs), and 48 of the 73 (65.8%) patients were male. While all73 patients received at least one cycle of NAC, 37 (50.7%) and 36 (49.3%) patients received doublet and triplet NAC regimen, respectively. Among the 73 patients, 50 (68.5%) and 20 (27.4%) patients showed tumor partial response (PR) and stable disease (SD), respectively. However, tumor response was not evaluated for 3 (4.1%) patients because 2 patients developed ileus and one patient missed assessment due to personal reasons. Of the 73 patients that had underwent NAC, 64 (87.7%) underwent complete resection with negative margins (R0), 5 (6.8%) underwent microscopic resection (R1) and 4 (5.5%) patients underwent resection with grossly positive margins (R2). While 51 (70.0%) patients received post-operative chemotherapy in our hospital, there was no record for 22 (30%) patients. Adjuvant chemotherapy (AC) regimen in 23 patients was the same as NAC. Eight(11%) patients received post-operative radiotherapy. Median follow-up period was 635 days (range 123 - 1962 days) with Aug 20, 2013as the cutoff date. At this time, 37 patients were still alive with 29 out of the 37 showing no documented progression.

**Table 1:** Patient and treatment characteristics

Characteristics	No. of patients	Proportion
**Gender**		
Male	48	65.8%
Female	25	34.2%
**Age at diagnosis**		
< 60	47	64.4%
≥60	26	35.6%
**NAC regimen**		
Doublet	37	50.7%
Triplet	36	49.3%
Clinical response to NAC (RECIST)^*^		
SD	20	27.4%
PR	50	68.5%
**Type of resection**		
R0	64	87.7%
R1	5	6.8%
R2	4	5.5%
**Graded pathologic response**		
Minor	40	54.8%
Moderate	19	26.0%
Major	14	19.2%
**N stage**		
N0	19	26.0%
N1	15	20.5%
N2	13	17.8%
N3	26	35.6%
**Lymph node metastasis ratio**		
NR0	27	37.0%
NR1	15	20.5%
NR2	19	26.0%
NR3	12	16.4%
**vascular invasion‡**		
Yes	19	26.0%
No	53	72.6%
**Nervous invasion§**		
Yes	8	11.0%
No	64	87.7%
**Adjuvant Chemotherapy║**		
Yes	51	70.0%
No	22	
**Postoperative Radiotherapy¶**		
Yes	8	11.0%
No	65	

* Tumor response to NAC was not evaluated for 3 patients; 2 of them developed ileus during treatment, and the other one missed assessment for personal reasons.

‡/§ vascular invasion and nervous invasion status were not reported for one patient.

║51 patients were administered post-surgery chemotherapy and had medical records in our hospital;The post-surgery chemotherapy records of 22 patients was not known.

¶ 8 patients received post-operative radiotherapy.

### Prognostic factors associated with disease-free survival

**Figure 1:**
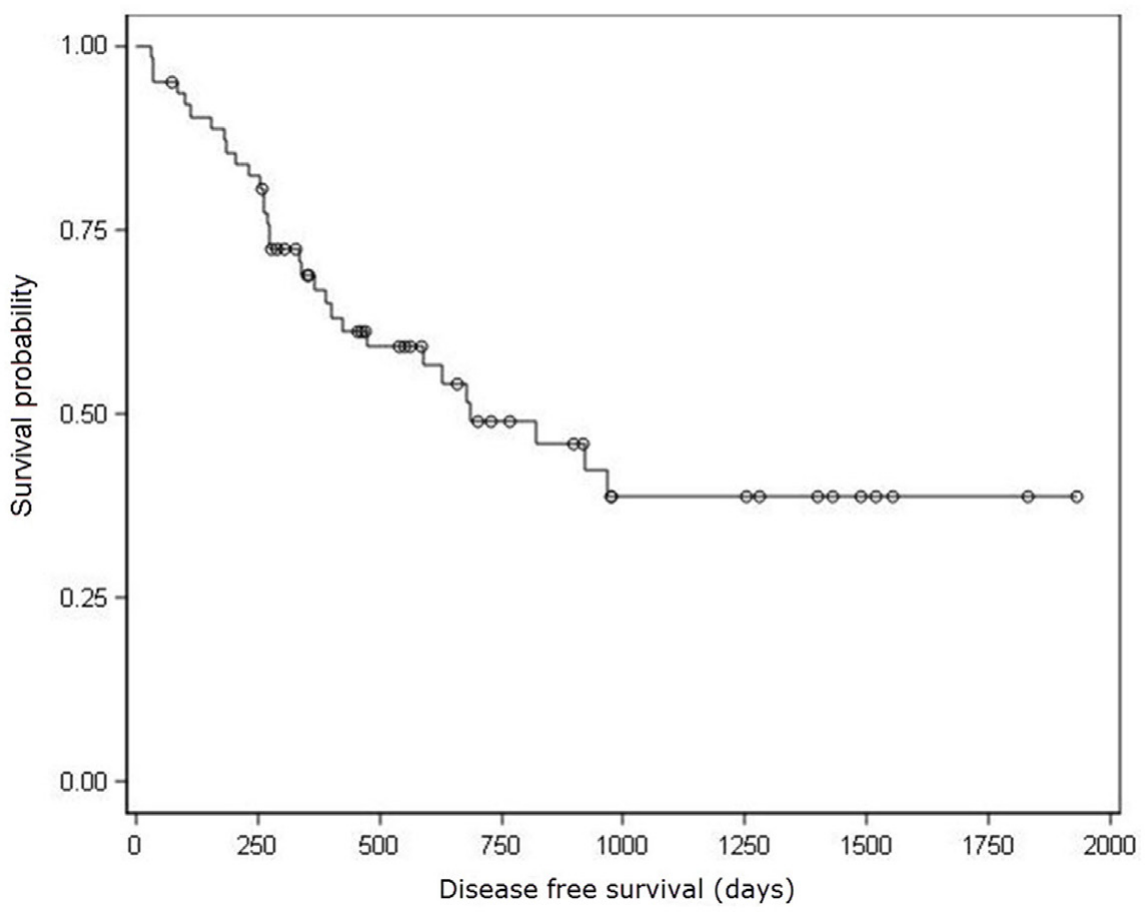
Kaplan–Meier analysis of disease free survival ○ Censored Patients. Median DFS for 64 patients that received R0 resection was 685 days.

Median DFS for 64 patients that received R0 resection was 685 days (Figure 1). Univariate analysis showed that nodal stages, lymph node metastasis ratio and nervous invasion were prognostic factors associated with DFS (Table 2). Patients with N0, N1 and N3 stages showed decreasing DFS rates (923, 630, and 263 days, respectively; P<0.0001; insufficient data to estimate DFS in N2 stage patients). Patients with lymph node metastasis ratiosNR0, NR1, NR2, and NR3also demonstrated decreasing DFS rates (923, 821, 679 and 267 days, respectively; P=0.0022). Patients with nervous invasion showed lower DFS (185 days) than patients without nervous invasion (821 days; P=0.0139).Furthermore, vascular invasion and elevated serum CA199 levels were marginally associated with shorter DFS (P = 0.0666 and P = 0.0613, respectively).

**Table 2:** Univariate prognostic factors analyses of disease-free and overall survival

Variable	DFS		P value	OS		P value
	No. of patients	Median		No. of patients	Median	
**Age at diagnosis**						
≥60	23	679	0.8901	26	878	0.4526
<60	41	658		47	953	
**Gender**						
Male	43	685	0.5737	48	878	0.9442
Female	21	630		25	930	
**CEA**						
Abnormal	13	426	0.5313	18	564	0.0400
Normal	46	821		50	1039	
**CA199**						
Abnormal	15	402	0.0613	19	624	0.0254
Normal	44	923		49	1165	
**CA724**						
Abnormal	14	304	0.1402	17	624	0.2505
Normal	40	630		46	878	
**Neoadjuvant chemotherapy regimen**						
Doublet	30	821	0.7547	37	878	0.3531
Triplet	34	679		36	1039	
**RECIST**						
SD	15	923	0.3229	20	806	0.3351
PR	47	630		50	930	
**Graded pathologic response**						
Minor	31	821	0.9908	40	752	0.0758
Moderate	19	591		19	1039	
Major	14	685		14	NE†	
**Resection Type**						
R0	64	685	NA*	64	1165	<0.0001
R1+R2	NA	NA*		9	452	
**Nodal stage**						
N3	19	263	<0.0001	26	550	<0.0001
N2	12	NE†		13	NE†	
N1	15	630		15	1165	
N0	18	923		19	NE†	
**Lymph node metastasis ratio**						
NR3	12	268	0.0022	12	604	0.0215
NR2	19	679		19	1624	
NR1	15	821		15	1165	
NR0	18	923		27	953	
**Vascular invasion**						
Yes	15	388	0.0666	19	604	0.0782
No	48	923		53	1431	
**Nervous invasion**						
Yes	5	185	0.0139	8	443.5	0.0003
No	58	821		64	1039	
**ifAC and NAC regimensaresimilar**						
Yes	21	679	0.7393	23	930	0.7334
No	43	821		50	878	

*NAs are not applicable; †NEs not estimated because of insufficient survival data.

Multivariate analysis by stepwise Cox model showed that post-treatment nodal stage, nervous invasion and serum CA199 levels were independent prognostic factors for DFS (Table 3). Higher N stages [N1 (HR =2.028, 95% CI = 0.604–6.808), N2 (HR = 0.812, 95%CI = 0.187-3.530), and N3 (HR = 7.044, 95% CI = 2.189-22.666), P=0.002], nervous invasion (HR = 3.647; 95% CI = 1.004-13.242; P=0.0492), elevated serumCA199(HR = 2.540; 95% CI = 1.044-6.176; P=0.0398) levels showed significant association with shorter DFS. In contrast, lymph node metastasis ratio and presence of vascular invasion were not associated with DFS according to multivariate analysis.

**Table 3:** Multivariate analysis of prognostic factors for disease-free survival by stepwise Cox model

Variable	Hazard Ratio	95% CI	P value
**Nervous invasion**			
Yes	3.647	(1.004 - 13.242)	0.0492
No	1		
**Nodal stage**			
N3	7.044	(2.189 - 22.666)	0.0020
N2	0.812	(0.187 - 3.530)	
N1	2.028	(0.604 - 6.808)	
N0	1	-	
**CA199**			
Abnormal	2.540	(1.044 - 6.176)	0.0398
Normal	1	-	

### Prognostic factors associated with overall survival

**Figure 2:**
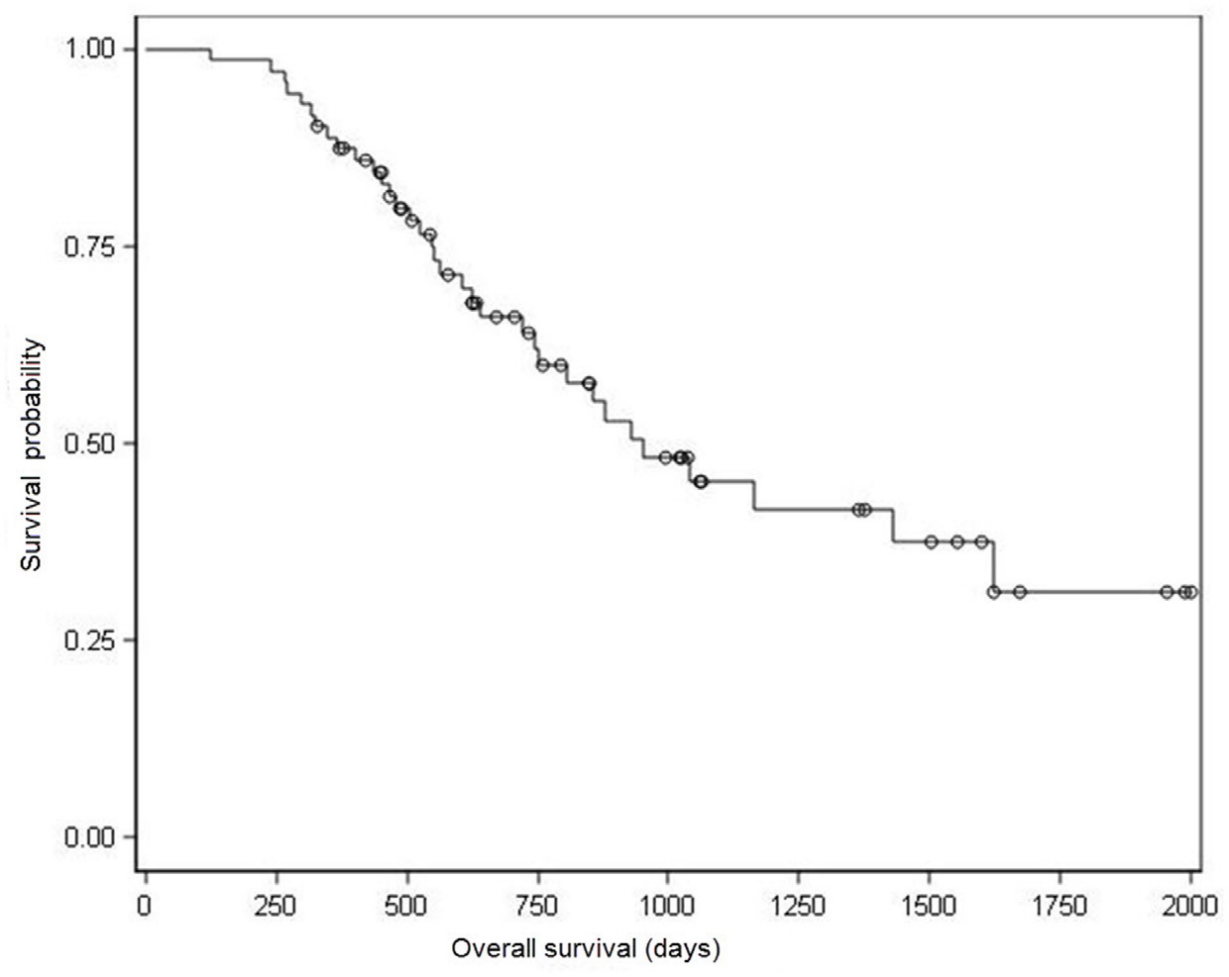
Kaplan–Meier analysis of overall survival ○ Censored Patients. Median OS for all of these 73 patients was 930 days.

Kaplan-Meier survival analysis showed a median OS of 930 days (Figure 2). Univariate analysis for OS showed that nodal stage (N), lymph node metastasis ratio (NR), nervous invasion, resection type and serum CEA/ CA199ratiowere prognostic indicators of OS, whereas, graded pathologic response and presence of vascular invasion showed marginal association with OS (Table 2).

Multivariate analyses showed that higher nodal stages [N1 (HR = 7.869, 95% CI = 2.191-28.266), N2 (HR = 0.761, 95% CI = 0.144-4.024) and N3 (HR = 2.923, 95% CI = 0.648-13.185); P = 0.0007], presence of nervous invasion (HR = 3.283; 95 % CI = 1.154-9.339; P = 0.0259) and non-radical resection (HR = 2.807; 95 % CI = 1.207-6.526; P = 0.0165) were independent prognostic factors for poor OS (Table 4).

**Table 4:** Multivariate analysis of prognostic factors for overall survival by the stepwise Cox’s model

Variable	Hazard Ratio	95% CI	P value
**Nodal stage**			
N3	7.869	(2.191, 28.266)	0.0007
N2	0.761	(0.144, 4.024)	
N1	2.923	(0.648, 13.185)	
N0	1	-	
**Nervous invasion**			
Yes	3.283	(1.154, 9.339)	0.0259
No	1	-	
**Resection type**			
R1+R2	2.807	(1.207, 6.526)	0.0165
R0	1	-	

## DISCUSSION

Patients with potentially resectable gastric cancer are treated with neoadjuvant chemotherapy to improve survival. However, a standard regimen is not clear. Several investigations have shown that patients that received NAC followed by surgery, age at diagnosis, post-treatment nodal status, diffuse-type histology, perineural invasion/vascular invasion and salvage surgery are associated with OS [10–13]. Our study demonstrated that clinical response (SD or PR) was not a significant prognosis factor for DFS or OS. One possible reason is that we enrolled only limited number of patients that underwent curative-intent surgery after NAC.

The prognostic value of pathologic response to neoadjuvant chemotherapy remains controversial although it has been investigated for various malignancies. Kurokawa Y *et al.* suggested that pathological response was a better surrogate endpoint than RECIST in neoadjuvant chemotherapy for gastric cancer [14]. In many studies, univariate analysis showed that pathologic response was a predictor of survival in GC patients after receiving neoadjuvant chemotherapy; but, multivariate analysis showed that it was not an independent predictor of OS [10, 15–18]. In this study, univariate analysis showed that pathologic response was marginally associated with OS, but not associated with DFS; multivariate analysis showed that it was not associated with both DFS and OS. Fujitani*et al.* showed that pathologic response was associated with OS in the subset of patients with nodal stages N0–1 [10].Therefore, further prospective studies with larger sample size are necessary to confirm the prognostic status of pathologic response in patients that undergo curative-intent surgery after NAC.

Metastatic lymph node ratio (NR) is an alternate prognostic factor instead of the number of lymph nodes (N in TNM staging) in GC because of the limited number of lymph nodes [18, 19]. Persiani R *et al.* showed that TRM (R means metastatic lymph node ratio) staging system had better prognostic power than the TNM system by reviewing 219 patients that underwent gastrectomy for node-positive (M0) cancer [20]. Posteraro B *et al.* retrospectively reviewed 110 patients that received curative-intent gastrectomy by the TRM staging and demonstrated that higher NR strongly predicted poor OS and DFS [21]. In the present study, NR was a prognostic factor for both DFS and OS in univariate analysis, but was in significant in multivariable analysis. However, post-treatment N stage showed association with DFS and OS in both univariable and multivariable analyses, consistent with the study by Fujitani *et al.*[10].Therefore, post-treatment N stage was a more reliable prognostic factor than metastatic lymph node ratio (NR) in locally advanced GC patients that received gastrectomy after NAC.

Changes in serum CA199 levels demonstrate therapeutic efficacy with increased serum CA199 levels indicating treatment failure or recurrence. Mohri *et al.* showed thatCA199 was an independent prognostic factor for OS in patients with metastatic gastric cancer [22]. Schauer *et al* demonstrated that serum CA199 levels predicted survival in patients with diffuse type gastric cancer after surgical treatment [23]. Zhu *et al* showed that serum CA199 was part of the prognostic index for patients with metastatic gastric cancer that received epirubicin (EPR)-containing triplet regimen as first-line treatment [24]. In our study, univariate analysis showed that elevated serumCA199 levels were associated with both shorter DFS and OS, but multivariate analysis showed that it was associated with poor DFS. This suggested that serum CA199 levels were strong predictors of long-term survival inGC patients.

Our findings are limited because this was a retrospective study conducted in a single institution with few select patients (73 study subjects) with different pre- and post-operative chemotherapeutic regimens. Larger multi-center prospective studies are necessary to confirm our findings.

In conclusion, our study demonstrates that post-treatment nodal stages, neural invasion and serum CA199 levels are associated with poor DFS. Moreover, post-treatment nodal stage, resection type and neural invasion status are independent prognostic factors for OS. This study also revealed that post-treatment N stage was a more reliable prognostic factor than metastatic lymph node ratio (NR) in locally advanced GC patients that received gastrectomy after NAC.

## MATERIALS AND METHODS

### Patients and treatment schedule

We retrospectively analyzed the outcomes of 73 consecutive patients with locally advanced gastric cancer that were treated with neoadjuvant chemotherapy followed by surgical resection between August 2007 and July 2012 at the National Cancer Center/Cancer Hospital, Chinese Academy of Medical Sciences & Peking Union Medical College, China. The procedures followed were in accordance with the ethical standards set by the independent ethnic committee of Cancer Hospital of Chinese Academy of Medical Sciences & Peking Union Medical College on human experimentation and the Helsinki Declaration. The patients were diagnosed with resectable advanced adenocarcinoma (gastric cancer) and completed at least one course of NAC with the tumor response evaluated by Response Evaluation Criteria in Solid Tumors (RECIST, version 1.0). All patients received gastrectomy, with D2 lymph-node dissection. The post-therapy node status including number of metastatic lymph nodes (N stage) and lymph node metastasis ratio (NR) were determined after examination. The extent of residual tumor was determined by the pathologist in our hospital to estimate the pathologic response to NAC. After surgery, patients received post-operative chemotherapy and/or radiotherapy based on the clinical status of individual patients. The patients followed up by regular clinic visits and phone calls.

### Statistical analysis

SAS statistical software 9.3 (SAS Institute Inc., Cary, NC, USA) was used for all statistical analyses. Overall survival (OS) was defined as the time from the date of initiation of NAC until death from any cause (event) or the last follow-up date (censored). Disease-free survival (DFS) was defined as the time from the date of surgery until local or distant relapse was detected (event) or the last follow-up date (censored). Kaplan Meier survival analysis was used to determine both DFS and OS.

Univariate analyses were performed by the log rank test for the following variables: age at diagnosis (≥ 60 vs. ≤ 60); gender (male vs. female); abnormal versus normal levels of serum CEA, CA199and CA724; Neoadjuvant chemotherapy regimen [doublet (Fluoropyrimidine + Platinum) versus triplet (Fluoropyrimidine + Platinum + Anthracyclines or Taxane)]; clinical response (partial response vs. stable disease); graded pathologic response (minor vs. moderate vs. major); resection type (R0 vs. R1+R2); Nodal stage [N0 (no metastasis), N1 (1-2 metastatic nodes), N2 (3-6 metastatic nodes), or N3 (7 or more metastatic nodes)]; lymph node metastasis ratio [number of positive nodes/number of nodes examined and classified as NR0 (0%), NR1 (<15%), NR2 (15-40%), or NR3 (>40%)];presence or absence of vascular invasion; presence or absence of nervous invasion; and if regimen of AC was the same as NAC or not.

Multivariate analysis of prognostic factors was performed by the stepwise Cox proportional hazard model with the variables identified as significant factors in the univariate analyses and hazard ratio (HR) and their95% confidence intervals (CIs) were calculated. A two-sided P < 0.05 was considered statistically significant.
